# Oligonuclear Metal Complexes with Schiff Base Ligands

**DOI:** 10.3390/ijms241311014

**Published:** 2023-07-03

**Authors:** Luca Rigamonti

**Affiliations:** Dipartimento di Scienze Chimiche e Geologiche, Università degli Studi di Modena e Reggio Emilia, Via G. Campi 103, 41125 Modena, Italy; luca.rigamonti@unimore.it; Tel.: +39-0592058646

As stated by two of the seven papers [1,2] published in the Special Issue entitled ‘Oligonuclear Metal Complexes with Schiff Base Ligands’ that I guest edited, the chemistry of Schiff bases began in 1864 due to the discovery made by Hugo Schiff [3], but even more than one and a half centuries after their first synthesis, Schiff bases continue to surprise inorganic chemists.

The condensation of a carbonyl moiety RR’C=O (R, R’ = alkyl or aryl groups for ketones, R’ = H for aldehydes) with a primary amine R”NH_2_ (R” = alkyl or aryl group) forms an iminic RR’C=NR” bond, referred to as azomethine or secondary aldimmine when R’ = H [4], and the lone pair of the iminic nitrogen atom, along with being basic, is able to act as a ligand toward metal centers [5]. C=N is usually incorporated into more articulated architectures in addition to other donor atoms, the so-called polydentate ligands [6], since metal complexes with macrocyclic and acyclic polydentate Schiff bases [7,8] show enhanced thermodynamic stability due to the chelating effect [9]. It is of course mandatory to name the most famous acyclic tetradentate N_2_O_2_ Schiff base *N*,*N*′-*bis*(salicylidene)ethylenediamine, H_2_salen, obtained via the condensation of two moles of salicylaldehyde, salH, and one mole of ethylenediamine, en, and known for almost one century [10]. Depending on the disposition of the donor atoms in the organic skeleton, Schiff base ligands can form mononuclear or oligonuclear metal complexes and, for example, the salen scaffold suitably hosts metal ions by occupying four coordination positions in a square planar fashion, thus preferably forming mononuclear species [11,12,13].

The aliphatic diamine en in H_2_salen can be replaced by the aromatic 1,2-phenylenediamine, phen, obtaining the more rigid tetradentate N_2_O_2_ H_2_salphen [14,15]. Janet Soleimannejad, E. Carolina Sañudo and co-workers suitably engineered this ligand by introducing cathecol and naphtol groups on salH in their paper entitled ‘Cathecol and Naphtol Groups in Salphen-Type Schiff Bases for the Preparation of Polynuclear Complexes’ [16]. Mononuclear copper(II) and cerium(IV) complexes could be suitably isolated, as well as a dinuclear iron(III) species with a bridging oxido ion, displaying a nearly linear Fe–O–Fe bridge with very strong antiferromagnetic coupling constant *J* = −255 cm^−1^ (–*J* **Ŝ**_1_·**Ŝ**_2_ formalism) between the two metal ions. A tetranuclear cluster with mixed-valence manganese(II)/(III) ions was also assembled when the ligand containing the cathecol fragments were employed, thanks to the two phenolato oxygen atoms in *ortho* position.

Longer spacers between the two salH moieties can be also employed, such as for example 1,3-diaminopropane [17,18], or even more articulated molecules like oxalyldihydrazide, as shown by Manas Sutradhar, Luísa M. D. R. S. Martins and co-workers in their contribution entitled ‘Aroylhydrazone Schiff Base Derived Cu(II) and V(V) Complexes: Efficient Catalysts towards Neat Microwave-Assisted Oxidation of Alcohols’ [19]. The presence of further nitrogen and oxygen donor atoms along with the Schiff base *N*′_1_,*N*′_2_-*bis*(2-hydroxybenzylidene)oxalohydrazide induces the formation of a hexanuclear copper(II) cluster and a dinuclear oxidovanadium(V) species. Both complexes were tested as homogeneous catalyst precursors for the microwave-assisted neat oxidation of primary or secondary alcohols to the corresponding carbonyl compounds (aldehydes for primary alcohols and ketones for secondary alcohols) under low-power (5–10 W) irradiation using aqueous *tert*-butyl hydroperoxide (TBHP) as an oxidizing agent without any added solvent. The copper(II) derivative exhibited higher catalytic activity than the vanadium(V) one for all of the tested substrates, and its performances were even better under milder reaction conditions than the reported aroylhydrazone copper(II) analogues [20].

The reactivity of the shortened salen-type ligands H_3_salmp, H_2_salmen and H_2_sal(*p*-X)ben with variable *para*-substituent on the central aromatic ring (X = *t*Bu, Me, H, F, Cl, CF_3_, NO_2_) towards the trivalent metal ions manganese(III) and iron(III) was instead presented in the research paper I co-authored entitled ‘Selective Formation, Reactivity, Redox and Magnetic Properties of MnIII and FeIII Dinuclear Complexes with Shortened Salen-Type Schiff Base Ligands’ [21]. The one-carbon bridge between the two iminic nitrogen atoms drives toward the selective formation of dinuclear complexes, together with hydrolytic C–N bond breakage in the case of iron(III). The antiferromagnetic (AFM) coupling constants *J* (−2*J* **Ŝ**_1_·**Ŝ**_2_ formalism) between the metal centers had values around −13 cm^−1^ for manganese(III) compounds, among the largest AFM coupling constants reported so far for dinuclear Mn^III^_2_ derivatives, while values between −3 and −10 cm^−1^ were obtained for iron(III) compounds.

Schiff bases derived from 2-hydroxy-5-methylisophthalaldehyde and histamine or 2-(2-aminoethyl)pyridine were instead employed by Magdalena Barwiolek, Anna Kaczmarek-Kędziera and co-workers to assemble dinuclear copper(II) derivatives and presented in their article entitled ‘Dinuclear Copper(II) Complexes with Schiff Bases Derived from 2-Hydroxy-5-Methylisophthalaldehyde and Histamine or 2-(2-Aminoethyl)pyridine and Their Application as Magnetic and Fluorescent Materials in Thin Film Deposition’ [22]. The complexes were studied for their magnetic properties, revealing AFM interactions between the two metal centers, and for their optical behavior, both in the bulk and deposited on Si(111) by spin coating, showing fluorescence between 489 and 509 and 460 and 464 nm for the compounds and the layers, respectively.

The last research paper by Catherine E. Housecroft and co-workers entitled ‘Schiff Base Ancillary Ligands in Bis(diimine) Copper(I) Dye-Sensitized Solar Cells’ reported an elegant surfaces-as-ligands, surfaces-as-complexes (SALSAC) approach [23] for obtaining the heteroleptic copper(I) complexes [Cu(L_anchor_)(L_ancillary_)]^+^ assembled on FTO-TiO_2_ electrodes and incorporated as dyes into *n*-type dye-sensitized solar cells (DSCs), where L_anchor_ is a phosphonic acid-functionalized 2,2′-bipyridyl anchoring ligand and L_ancillary_ is a 6,6′-dimethyl-2,2′-bipyridyl ligand bearing *N*-arylmethaniminyl substituents [24]. Comparisons between performances of DSCs containing the Schiff base complexes with those sensitized by analogous dyes lacking the imine bond indicate that the latter prevents efficient electron transfer across the dye.

The first review of the Special Issue by Demetrios I. Tzimopoulos, Malgorzata Holynska, Spyros P. Perlepes and co-workers entitled ‘Oligonuclear Actinoid Complexes with Schiff Bases as Ligands—Older Achievements and Recent Progress’ deals with the coordination chemistry of Schiff bases with actinoid (5*f* elements) ions [2]. This is an emerging area in recent years, compared to the more mature chemistry of transition metals and lanthanides. The handling and recycling of nuclear materials, the recovery of *trans*-{U^VI^O_2_}^2+^ from the oceans and the catalytic activation of small molecules are among the possible applications of Schiff base ligands with actinides. This review thoroughly covers aspects of synthetic chemistry, reactivity, and the properties of dinuclear and oligonuclear actinoid complexes based on Schiff base ligands within the last 20 years in an extensive way and with clear presentation.

The second review in this Special Issue by Barbara Miroslaw entitled ‘Homo- and Hetero-Oligonuclear Complexes of Platinum Group Metals (PGM) Coordinated by Imine Schiff Base Ligands’ presents the most recent concepts on the construction of homo- and hetero-oligonuclear Schiff base coordination compounds of PGMs (iridium, osmium, palladium, platinum, rhodium, and ruthenium) [1]. Ranging from mono- to poly-dentate Schiff bases, this review elegantly shows the potency of these ligands to form variegate coordination and metallorganic architectures with a very efficient visual approach, and always focusing on the potential applications of the obtained compounds.

All papers and reviews of the Special Issue have added fundamental knowledge in the field of Schiff bases and related oligonuclear metal complexes. I personally thank the authors for participating with their extraordinary articles, which are worthy of reading and being used as references in the ongoing research on Schiff base complexes.
